# The Surgery of M. Dieffenbach

**Published:** 1841-01

**Authors:** 


### ART. XII.

*La Chirurgie de M. Dieffenbach.* Par CHARLES PHILLIPS. Première Partie, avec quatre Planches.—*Berlin,* 1840. 8vo, pp. 200.

*The Surgery of M. Dieffenbach.* By CHARLES PHILLIPS. First Part, with four Plates.—*Berlin,* 1840.

THIS short treatise, written by a gentleman who, as M. Dieffenbach's assistant, had ample opportunities of observing closely his practice, describes the method of operation and the general results in certain subjects of surgery to which M. Dieffenbach has more particularly given his attention. We shall consider these subjects separately, briefly dwelling on any new features that the method of operation or the after-treatment may present.

The first few pages are devoted to the description of Professor Jungken's operations for staphyloma and artificial pupil. The former differs in no degree from that usually followed by all surgeons, namely, removal of the projecting cornea. In making an artificial pupil, the professor introduces a knife across the cornea, which he freely incises; the aqueous humour being expelled and the iris protruded, he seizes a portion of the iris with a fine hook and cuts it off with scissors. This mode of operation has the advantage of preventing the adhesion of the iris to the cornea, which commonly takes place when the former membrane is made to protrude through a small opening in the latter.

His boldness and success in advancing orthopedic surgery, one of the most important improvements that has been made in the modern treatment of disease, would have alone rendered the author's name celebrated. "Dieffenbach," says Mr. Phillips, "has, up to this time, operated on 300 distortions of the foot, sixty cases of wry-neck, a great number of contracted arms, hips, and knees. He has reduced a dislocation of two years' standing, and cured eighteen* cases of strabismus. In all these cases he has never experienced any considerable hemorrhage or caused lesion of any of the great nerves or blood-vessels."

In treating the various forms of talipes, he has divided all the tendons that pass from the leg to the foot; and, not content with a single operation, he repeats it until the deformity disappears: in one case the tendo achillis was divided twenty times before a complete cure was obtained. In valgus, the peronei muscles and extensors of the toes; in varus, the tendo achillis, flexors of the toes and of the great toe, the plantar fascia, tibialis anticus and extensors of the toes, have all been divided at the same time. The subcutaneous section of muscles has also been applied to the reduction of a dislocation of the shoulder.† The new capsule of the joint, the tendons of the supra and infra spinatus and the latissimus dorsi required to be cut across before the replacement of the bone in its proper situation could be effected. We think it doubtful how far such practice is to be recommended. In a dislocation of two years' standing, the head of the bone has formed for itself a new articulation; the muscles have adapted themselves to the displacement, and motion has become tolerably perfect; the former articulating surface of the bone has also become altered in form. These considerations would make us hesitate before we recommended a patient to submit to the risk of so severe an operation as dividing muscles deeply situated in the axilla would be, with the prospect of imperfect success. The tendons of the hip-joint and knee have been cut across in contractions of these articulations with a most favorable result; and cases are mentioned in which, when even anchylosis of the knee-joint had occurred, and the osseous union was broken up by the forcible extension of the joint after division of the tendons, the patient recovered a considerable degree of motion in the limb.

We have already noticed in our Journal the operations for strabismus.

Rupture of the perineum is the next subject noticed, and, in the treatment of this injury, cases of extraordinary success are mentioned. Dieffenbach attempts to unite these lacerations immediately after their occurrence, when he has the opportunity. In cases of older date, he is of course obliged to pare the edges of the divided parts before bringing them together. The union is effected by means of a great number of interrupted sutures placed very deeply: the twisted suture is occasionally used. The plan of treatment by operation is modified according to the variety of the case. He considers the success he obtains is not attributable to any peculiar mode of operation, but to the after-treatment. After the operation the patient is placed on her back in bed: the legs are not tied together, as is the usual practice; the secretions of the vagina that cannot but take place immediately after delivery, and when so severe an

* Since this was written he has operated on hundreds. See our last Number, p. 570.

† See our last Number, p. 566.

accident has occurred, instead of being allowed to remain in that cavity, and passing between and irritating the edges of the wound, are cleared away by frequent injections of water. Opium is administered to produce constipation, and at the end of seven or eight days an injection of oil is given: an evacuation having been procured by this means, constipation is again produced by the same medicine. By this treatment a great part of the lacerated surfaces will be united. Should any unnatural opening remain, it is touched with tincture of cantharides and argent. nitrat. In some of the cases a fistulous opening remained between the rectum and vagina: it was allowed to contract as far as it would naturally; the edges were then pared, brought together by the interrupted suture, and the same after-treatment pursued as is above indicated: if one operation is not successful it is repeated. This mode of treatment is evidently a great improvement on the usual method of tying the legs together, pressing the lacerated edges of the wound one against the other, and keeping them constantly bathed in the irritating secretions of the vagina and rectum.

There is nothing peculiar in Dieffenbach's mode of operation for fissures of the palate. After paring the edges of the fissure, he introduces small needles from behind forwards by means of a *porte-aiguille.* The ligatures are made of lead; the great recommendation of which he seems to think is, that the operator is enabled to tighten them without introducing his fingers into the mouth. It appears to us that this is rather an objection to metallic ligatures. The fingers of a careful operator would certainly be much less likely to irritate the patient than the instruments that must be used in this operation.

Tumours of the face and operations on the upper and lower jaw have occupied the attention of Dieffenbach to a considerable degree, but we cannot commend his practice in this department of surgery. He does not want boldness to execute, but there is a great want of judgment in the choice of the cases submitted to operations: all sorts of tumours, whether malignant or otherwise, are mentioned as being attacked by the knife. Now it cannot be doubted that the removal of the whole or portions of the upper or lower jaw are among the most successful of surgical operations, when undertaken in proper cases; but it is only to tumours of slow growth, firm consistence and benign character, that operation is applicable; and we cannot but think it a matter of cruelty, and perfectly useless, to subject a patient to these severe operations, when, from the nature of the disease, we may predicate an almost certain return. Such is the case in many of Dieffenbach's operations; and repeated removals of portions of the jaws, together with free applications of the actual cautery, have failed to eradicate the disease. Dieffenbach recommends, in removing the upper jaw, to make incisions in the skin, commencing from the zygoma inwards towards the upper part of the nose, passing under the lower eyelid, and another incision down the centre of the nose and through the upper lip: the flap of integument is then dissected back. We think that preferable incisions, and not likely to produce so much deformity, are those carried from the prominence of the malar bone to the angle of the mouth, and by the side of the nose, round the ala, and through the centre of the upper lip. The flap of skin is easily raised; sufficient space is given for the future steps of the operation; the Stenonian duct is avoided, and but few branches of the portio dura are wounded. Dieffenbach removes the bones by means of small saws, strong scissors, &c., and applies the actual cautery most freely to the cavity. These instruments are far inferior to the strong cutting forceps, and the application of the actual cautery is a piece of needless cruelty. No operation of this kind should be undertaken, either where the disease is such that it has contaminated the surrounding parts, or where it cannot be entirely removed by cutting in the healthy structures: hence the perfect inutility of the cautery.

In plastic operations, Dieffenbach's practice is somewhat different from that usually pursued. In restoration of the whole of the nose, he borrows the skin from the hairy scalp, and connects it with the part to which it is to be transplanted by means of a long narrow pedicle down the centre of the forehead. The object of this mode of incision is to prevent the deformity that a large cicatrice in the forehead produces. The hairs soon cease to grow when the skin is transplanted to its new situation. In restoration of the ala of the nose, a very curious operation is performed. A central incision is made down the nose, and the skin is dissected off on each side from the bones, on the sound side, carrying with it the cartilage of the ala; the central cartilage is thus exposed, and out of this a triangular piece, the apex downwards, is cut; a similar piece is cut from above the ala on the sound side of the nose, which is by these means shortened to a sufficient degree to correspond with the shortened ala on the diseased side. Of course by such an operation the form of the nose is much altered, and a most determined "nez retroussé" is formed. We cannot but think the transplantation of a portion of the cheek to supply the deficiency in the ala is a much easier and less painful operation, and less likely to alter the general contour of the features. Several other cases of restoration of the eyelids, point of the nose, &c., are mentioned, which do not present any great interest, and for an account of which we must refer our readers to the work itself.

The latter pages of this little book are occupied by a number of unconnected surgical cases, for which operations have been performed. Among these, a new way of treating prolapsus uteri is mentioned. When this disease is very well marked, and the greater part of the uterus prolapses through the os externum, Dieffenbach, first having reduced the prolapsus, introduces a red-hot iron into the vagina and cauterizes the lower part of it and the os externum. When the sloughs separate, union takes place between the sides of the vagina and the labia, and a barrier is formed which completely prevents the prolapsus descending. This is rather a severe proceeding, and there are few patients who would be induced to submit to such an operation; and, luckily, it is rare that the prolapsus is to such an extent that relief cannot be afforded by properly constructed pessaries.

In strictures of the urethra, where retention exists, and a catheter cannot be passed through the stricture, Dieffenbach's practice is to cut transversely across the urethra, posterior to the stricture. He observes that the operation of cutting on a catheter in the middle line is a very difficult one. We should think he never could have seen practised or have attempted to perform this latter operation. By placing the finger in the rectum and passing a sharp bistoury, with the back towards the bowels, guided by the finger up towards the prostate, the dilated portion of the urethra can be most easily opened in the middle line, and the knife, being drawn forwards towards a catheter, introduced as far as it can be into the urethra anterior to the stricture, divides every obstruction to the immediate introduction of the instrument into the bladder. This operation will be found a very successful one, and, after a few weeks, the urethra will be restored to a very healthy state, requiring only the occasional introduction of bougies.

We shall close our observations with a case of cancer of the rectum, in which Dieffenbach attempted to extirpate the diseased parts. He first made a free incision at the anterior and posterior edge of the bowel; he seized the diseased parts with a vulsellum, and, having brought them towards the anus, removed them with scissors. We can only remark, that if this was a case of true cancer such a proceeding is worse than useless.

The perusal of this book will leave no doubt of Dieffenbach's great skill as an operator, and of the numerous original views and plans he has introduced in his peculiar department of science; but an impression is also left on the mind of the reader that he is deficient in carefully distinguishing those cases in which operation will be really beneficial, and that he excels more as a skilful manipulator than as a sound surgeon.

---

